# Impaired Lipid and Glucose Homeostasis in Hexabromocyclododecane-Exposed Mice Fed a High-Fat Diet

**DOI:** 10.1289/ehp.1307421

**Published:** 2014-01-07

**Authors:** Rie Yanagisawa, Eiko Koike, Tin-Tin Win-Shwe, Megumi Yamamoto, Hirohisa Takano

**Affiliations:** 1 Center for Environmental Health Sciences, National Institute for Environmental Studies, Tsukuba, Japan; 2 Department of Basic Medical Sciences, National Institute for Minamata Disease, Minamata, Japan; 3 Graduate School of Engineering, Kyoto University, Kyoto, Japan

## Abstract

Background: Hexabromocyclododecane (HBCD) is an additive flame retardant used in the textile industry and in polystyrene foam manufacturing. Because of its lipophilicity and persistency, HBCD accumulates in adipose tissue and thus has the potential of causing metabolic disorders through disruption of lipid and glucose homeostasis. However, the association between HBCD and obesity remains unclear.

Objectives: We investigated whether exposure to HBCD contributes to initiation and progression of obesity and related metabolic dysfunction in mice fed a normal diet (ND) or a high-fat diet (HFD).

Methods: Male C57BL/6J mice were fed a HFD (62.2 kcal% fat) or a ND and treated orally with HBCD (0, 1.75, 35, or 700 μg/kg body weight) weekly from 6 to 20 weeks of age. We examined body weight, liver weight, blood biochemistry, histopathological changes, and gene expression profiles in the liver and adipose tissue.

Results: In HFD-fed mice, body and liver weight were markedly increased in mice treated with the high (700 μg/kg) and medium (35 μg/kg) doses of HBCD compared with vehicle. This effect was more prominent in the high-dose group. These increases were paralleled by increases in random blood glucose and insulin levels and enhancement of microvesicular steatosis and macrophage accumulation in adipose tissue. HBCD-treated HFD-fed mice also had increased mRNA levels of *Pparg* (peroxisome proliferator-activated receptor-γ) in the liver and decreased mRNA levels of *Glut4* (glucose transporter 4) in adipose tissue compared with vehicle-treated HFD-fed mice.

Conclusions: Our findings suggest that HBCD may contribute to enhancement of diet-induced body weight gain and metabolic dysfunction through disruption of lipid and glucose homeostasis, resulting in accelerated progression of obesity.

Citation: Yanagisawa R, Koike E, Win-Shwe TT, Yamamoto M, Takano H. 2014. Impaired lipid and glucose homeostasis in hexabromocyclododecane-exposed mice fed a high-fat diet. Environ Health Perspect 122:277–283; http://dx.doi.org/10.1289/ehp.1307421

## Introduction

Hexabromocyclododecane (HBCD) (for chemical structure, see Supplemental Material, Figure S1) is a brominated flame retardant (BFR) that is incorporated into plastics, electrical and electronic products, textiles, and other materials to decrease flammability (de Wit 2002). HBCD is a ubiquitous and global environmental contaminant. It is highly bioaccumulative because of its persistent and lipophilic properties (Covaci et al. 2006). Human exposure to HBCD occurs mainly through a combination of diet, ingestion of indoor dust, and inhalation of indoor air (Harrad et al. 2010; Roosens et al. 2009). Diet is considered the most important source, particularly in humans consuming large quantities of fish that contain relatively high levels of HBCD (Janák et al. 2005; Xian et al. 2008). HBCD has been detected not only in human blood and breast milk (Fängström et al. 2008; Weiss et al. 2006) but also in human adipose tissue (Covaci et al. 2008; Malarvannan et al. 2013). Various classes of organohalogenated compounds, including HBCD isomers, have been detected in visceral and subcutaneous abdominal fat of obese individuals in Belgium (Malarvannan et al. 2013).

From a toxicological perspective, animal studies have shown that HBCD is a hepatic enzyme inducer (Germer et al. 2006), a developmental neurotoxicant (Eriksson et al. 2006), and an endocrine disruptor (van der Ven et al. 2006). The results of a recent study (Koike et al. 2012) suggested that some BFRs, including HBCD, activate immune cells and subsequently enhance immune/allergic responses. Cantón et al. (2008) reported that subacute HBCD exposure down-regulates cholesterol biosynthesis- and lipid metabolism–related gene expression in female rat liver, but up-regulates drug-metabolizing enzymes such as cytochrome P450 3A in male rat liver. These results suggest that exposure to HBCD may disrupt the metabolic, endocrine, and immune systems, resulting in impaired lipid and glucose homeostasis; however, the biological effects of HBCD have not been clarified.

The prevalence of obesity in adults and children has markedly increased worldwide over the past several decades (Ogden et al. 2002). Imbalance between caloric intake and expenditure is considered a key cause of the obesity epidemic, but there is emerging evidence that exposure to environmental chemicals may also be an important contributor (Grün and Blumberg 2007; Newbold 2010). Janesick and Blumberg (2011) recently reported that environmental chemicals such as persistent organic pollutants (POPs) may play an important role in modulating the balance between energy intake and expenditure. Serum levels of POPs, such as polychlorinated biphenyls (PCBs), polychlorinated dibenzo-*p*-dioxins, polychlorinated dibenzofurans, and organochlorine pesticides may be associated with body mass index, elevated triglyceride levels, abdominal obesity, and cardiovascular diseases (Airaksinen et al. 2011; Ha et al. 2007; Lee et al. 2012; Uemura et al. 2009). In animal studies, POPs—including 2,3,7,8-tetrachlorodibenzo-*p*-dioxin (TCDD), 1,2,3,4,7,8-hexachlorodibenzo-*p*-dioxin, 3,3´,4,4´-tetrachlorobiphenyl (PCB-77), 3,3´,4,4´,5-pentachlorobiphenyl (PCB-126), 2,2´,4,4´,5,5´-hexachlorobiphenyl (PCB-153), and POP mixtures in crude salmon oil—have been associated with body weight gain, insulin resistance, abdominal obesity, hepatosteatosis, and atherosclerosis (Arsenescu et al. 2008; Ruzzin et al. 2010). “Obesogens” are hypothesized to be environmental chemicals that promote obesity directly by increasing adipocyte size and/or number, or indirectly by altering metabolic homeostasis or interfering with regulation of appetite and satiety, suggesting that environmental chemicals can regulate lipid metabolism and adipogenesis, and thus promote obesity (Baillie Hamilton 2002; Grün and Blumberg 2007; Newbold 2010). POPs such as TCDD and PCBs have generally been decreasing both in the environment and in humans during the past few decades because of the decline in the use and production of these compounds (Jones and de Voogt 1999). BFRs, including HBCD, have been globally restricted by the *Stockholm Convention on Persistent Organic Pollutants* (Stockholm Convention 2010); however, BFRs continue to leach from existing products and to be disposed of in landfills. In addition, the effects of HBCD on obesity and obesity-related metabolic disorders remain poorly understood.

High-fat diet (HFD) is a major cause of obesity and is related to the development of cardiovascular disease and diabetes. HFD may also contribute to the development of systemic inflammation and insulin resistance in these diseases (Grundy 2004; Kahn and Flier 2000). Dietary fat also plays an important role in modulating the metabolism and toxicity of environmental chemicals (Yu 2000). However, the relationship between dietary fat and HBCD has not been examined. In the present study, we hypothesized that individuals with diet-induced obesity may be more susceptible to HBCD due to its lipophilic nature and high persistency, leading to metabolic dysfunction via disruption of lipid and glucose homeostasis.

The aim of this study was to determine whether exposure to HBCD induces and/or enhances obesity and metabolic disorders through the disruption of lipid and glucose metabolism in the liver and adipose tissue in mice fed a normal diet (ND) or an HFD.

## Materials and Methods

*Animals*. Five-week-old male C57BL/6JJcl mice (*n* = 68) were purchased from Japan Clea Co. (Tokyo, Japan) and used for the experiments. Mice were housed individually in polycarbonate cages with wood-chip bedding in controlled conditions (12-hr light/dark cycle, 22–26°C, and 40–69% humidity). Food and tap water were provided *ad libitum*. Body weights at the start of the study were 21.4 ± 0.14 g, with no differences between groups. At 5 weeks of age, mice were randomly divided into eight groups (*n* = 46; 5–6 animals/group), with four groups fed HFD-60 (Oriental Yeast Co. Ltd., Tokyo, Japan) containing 62.2 kcal% fat, and the other four groups fed a normal diet (ND; AIN-93M; Oriental Yeast Co. Ltd.) (for composition of both diets, see Supplemental Material, Table S1).

HBCD (Sigma-Aldrich Co., St. Louis, MO, USA) was dissolved in acetone (Nacalai Tesque Inc., Kyoto, Japan) and diluted with olive oil (Nacalai Tesque). The tolerable daily intake of HBCD is 100 μg/kg body weight (BW)/day (equivalent to a dose of 700 μg/kg BW/week) (Saito et al. 2008). Therefore, we used three doses of HBCD: 1.75 (low; L-HBCD), 35 (medium; M-HBCD), and 700 (high; H-HBCD) μg/kg BW/week. Vehicle (olive oil containing 0.5% acetone) was used as the control. Mice were dosed with vehicle or HBCD by oral gavage once each week from 6 weeks to 20 weeks of age, for a total of 15 treatments. Mice were weighed weekly at the time of dosing. Daily water and food intake were monitored in all groups at the ages of 6, 10, 15, and 20 weeks. All procedures were approved by the Institutional Review Board of the National Institute for Environmental Studies and the National Institute for Minamata Disease. Animals were treated humanely and with regard for alleviation of suffering.

*Biochemical tests in serum and analysis of hepatic lipid*. At 20 weeks of age, all mice were euthanized under diethyl ether anesthesia 24 hr after the final administration of vehicle or HBCD. The chest and abdominal walls were opened, and blood was retrieved by cardiac puncture. Serum was stored at −80°C until use. Serum levels of aspartate aminotransferase (AST), alanine aminotransferase (ALT), total cholesterol (T-Cho), triglyceride (TG), and glucose were measured using the SPOTCHEM EZ SP-4430 analyzer (ARKRAY Inc., Kyoto, Japan). Hepatic lipids were extracted according to the Folch method, and hepatic T-Cho and TG contents were measured in four groups (ND + vehicle, ND + H-HBCD, HFD + vehicle, and HFD + H-HBCD; 5–6 animals per group) using the enzyme assay from Skylight Biotech Inc. (Akita, Japan). Serum insulin levels were measured by ELISA (Mouse Insulin ELISA KIT; Shibayagi Co., Shibukawa, Japan) in these same groups.

*Histopathological examination*. Liver and epididymal adipose tissues from all animals in the ND + vehicle, ND + H-HBCD, HFD + vehicle, and HFD + H-HBCD groups (5–6 animals per group) were fixed in 10% phosphate-buffered formalin (pH 7.4). Tissues were embedded in paraffin and cut into 4-μm-thick slices. Liver sections were stained with hematoxylin and eosin. The degree of the fatty change in the liver was evaluated using an Olympus AX80 microscope (Olympus Corp., Tokyo, Japan) in a blinded fashion.

Macrophages in the adipose tissue were detected by immunohistochemistry. Adipose tissue sections were treated with inactivated endogenous peroxidase buffer using 3% hydrogen peroxide followed by normal goat serum (5%) for 1 hr at room temperature to decrease nonspecific staining. The sections were then incubated in anti-F4/80 antibody (1:1000; Abcam, Cambridge, UK) overnight at 4°C. Immunohistochemical reactions were performed using SignalStain Boost IHC Detection Reagent and SignalStain DAB Substrates Kit (both from Cell Signaling Technology Inc., Danvers, MA, USA). Slides were counterstained with Mayer’s hematoxylin and mounted. The F4/80-positive cells in adipose tissue were evaluated using an Olympus AX80 microscope in a blinded fashion.

*Real-time reverse transcription polymerase chain reaction (RT-PCR) analysis*. Total RNA was extracted from liver epididymal adipose tissue of four groups of mice (ND + vehicle, ND + H-HBCD, HFD + vehicle, and HFD + H-HBCD; 5–6 animals/group) using the RNeasy Lipid Tissue Mini Kit (Qiagen, Hilden, Germany). The total RNA concentration was assessed spectrophotometrically with a NanoDrop spectrometer (Thermo Scientific, Wilmington, DE, USA). Total RNA was reverse transcribed to cDNA using a High-Capacity RNA-to-cDNA™ Kit (Applied Biosystems, Foster City, CA, USA). mRNA expression was quantified using the StepOne Plus™ Real-time PCR System (Applied Biosystems). RT-PCR was then performed at 50°C for 2 min, 95°C for 10 min, 95°C for 15 sec, and 60°C for 1 min, with the last two steps repeated for 40 cycles. Data were analyzed by the critical threshold (ΔC_T_) and the comparative critical threshold (ΔΔC_T_) methods using StepOne Plus™ Software version 2.2.2. The relative intensity was normalized to an endogenous control gene (hypoxanthine phosphoribosyltransferase 1; *Hprt1*). TaqMan probes and pairs for target and *Hprt1* genes (Applied Biosystems) are listed in Supplemental Material, Table S2.

*Glucose and insulin tolerance tests*. In a separate experiment, all mice in four groups (ND + vehicle, ND + H-HBCD, HFD + vehicle, and HFD + H-HBCD; *n* = 22; 5–6 animals/group) were fasted for 16 hr or 4 hr prior to performance of a glucose tolerance test (GTT) or insulin tolerance test (ITT), respectively, and blood was collected from the tail vein. For the GTT and ITT, respectively, mice were injected with d-glucose (2 g/kg *per os*; Wako Pure Chemical Industries, Osaka, Japan) at 17 weeks of age, or human insulin (0.75 U/kg, by intraperitoneal injection; Sigma-Aldrich) at 18 weeks of age. Blood glucose levels were measured with a blood glucose meter (Glutest Neo Super; Sanwa Kagaku Kenkyusho Co., Nagoya, Japan) at 0, 20, 40, 60, 90, and 120 min. Total values for the area under the curve (AUC; arbitrary units) were obtained without reference to baseline values. Therefore, the area below the observed levels was calculated.

*Statistical analysis*. Data are expressed as mean ± SE. The significance of variation among different groups was determined by one-way analysis of variance (ANOVA) or Kruskal–Wallis analysis using Ekuseru-Toukei 2010 statistical software (Social Survey Research Information Co., Tokyo, Japan). Differences between the experimental and control groups were determined by Dunnett’s multiple comparison test or Steel’s multiple comparison test; *p* < 0.05 was considered statistically significant.

## Results

*HBCD enhances HFD-induced weight gain and hepatic steatosis*. We observed an increase in body and liver weight in HFD-fed mice at 20 weeks of age compared with ND-fed mice (see Supplemental Material, Table S3). In addition, mice in the HFD + M-HBCD and HFD + H-HBCD groups showed markedly increased body and liver weight compared with vehicle-treated ND and HFD mice. In HBCD-treated HFD-fed mice, significant body weight gain was observed beginning at 15 weeks of age (see Supplemental Material, Figure S2). In contrast, we observed no alterations in body and liver weight in ND-fed mice with or without HBCD treatment. Food and water intake showed no significant differences between HBCD- and vehicle-treated groups fed either diet (data not shown).

**Figure 1:**
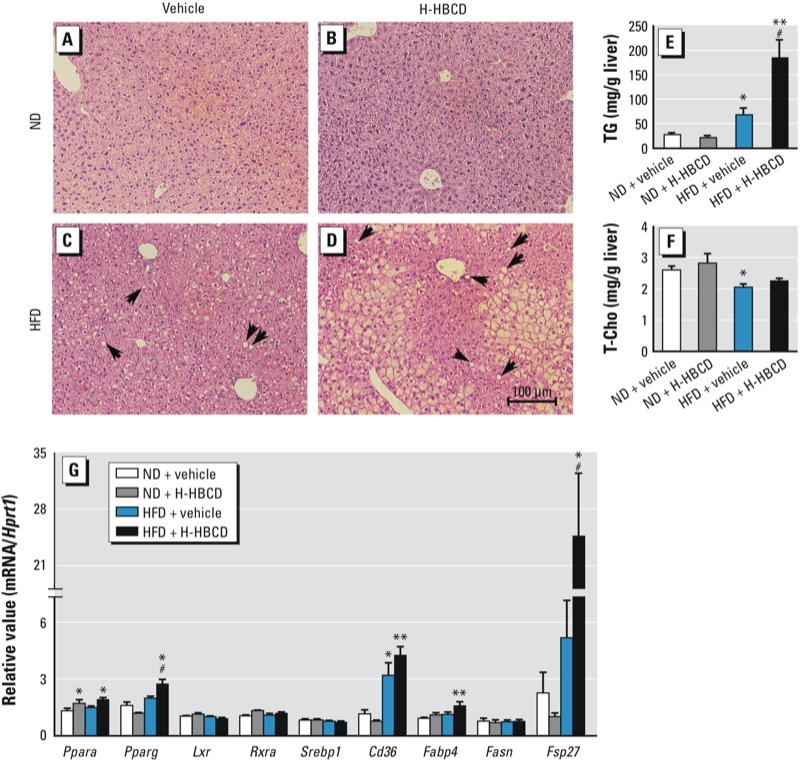
Histopathological findings, TG and T-Cho levels, and expression of lipid metabolism–related genes in the liver 24 hr after the final administration of vehicle or HBCD. Histopathological alterations were evaluated using hematoxylin and eosin staining. (*A*) ND + vehicle group. (*B*) ND + H‑HBCD (700 μg/kg/week). (*C*) HFD + vehicle. (*D*) HBCD + H‑HBCD. Arrows (*C,D*) indicate microvesicular steatosis. Hepatic TG (*E*) and T‑Cho (*F*) contents were measured by enzyme assay. (*G*) mRNA levels of *Ppara*, *Pparg*, *Lxr*, *Rxra*, *Srebp1*, *Cd36*, *Fabp4*, *Fasn*, and *Fsp27 *in the liver evaluated by real-time RT‑PCR. Data were are expressed as mean ± SE of 5–6 animals/group. Data were analyzed by ANOVA followed by Dunnett’s test or by Kruskal-Wallis test followed by Steel’s test. *p < 0.05, and **p < 0.01 compared with ND + vehicle. #*p *< 0.05 compared with HFD + vehicle.

In the histological analysis of the liver, we detected microvesicular steatosis in HFD-fed mice (Figure 1). In addition, HFD + H-HBCD resulted in development of severe microvesicular fatty changes, hepatocyte ballooning, and accumulation of hepatic TG (Figure 1D,E). T-Cho levels in liver tissues were lower in the HFD + vehicle group than in the ND + vehicle group (Figure 1F). Although serum T-Cho levels were higher in HFD-fed mice than in ND-fed mice, HBCD exposure had no significant effect in either group (see Supplemental Material, Table S3). Serum ALT levels were significantly higher in the HFD + M-HBCD and HFD + H-HBCD groups than in the ND + vehicle group. We noted no remarkable differences in serum TG levels.

Next, to elucidate the mechanisms of HBCD-induced hepatic steatosis in HFD-fed mice, we examined expression of lipid metabolism-related genes in the liver (Figure 1G). HFD + H-HBCD mice had significantly elevated mRNA levels of *Pparg* (peroxisome proliferator-activated receptor-γ) compared with ND + vehicle and HFD + vehicle groups (*p* < 0.05). Expression of PPARγ target genes, such as *Cd36* and *Fabp4* (fatty acid binding protein 4), increased in HFD + H-HBCD mice compared with ND + vehicle mice. *Fsp27* (cell death-inducing DFFA-like effector c) mRNA was significantly greater in the HFD + H-HBCD group than in the HFD + vehicle group (*p* < 0.05). H-HBCD induced *Ppara* mRNA in mice fed both diets, but we observed no changes in mRNA levels of *Lxr* (liver X receptor), *Rxr* (retinoid X receptor), *Srebp1* (sterol regulatory element binding transcription factor 1), or *Fasn* (fatty acid synthase) in either group.

**Figure 2:**
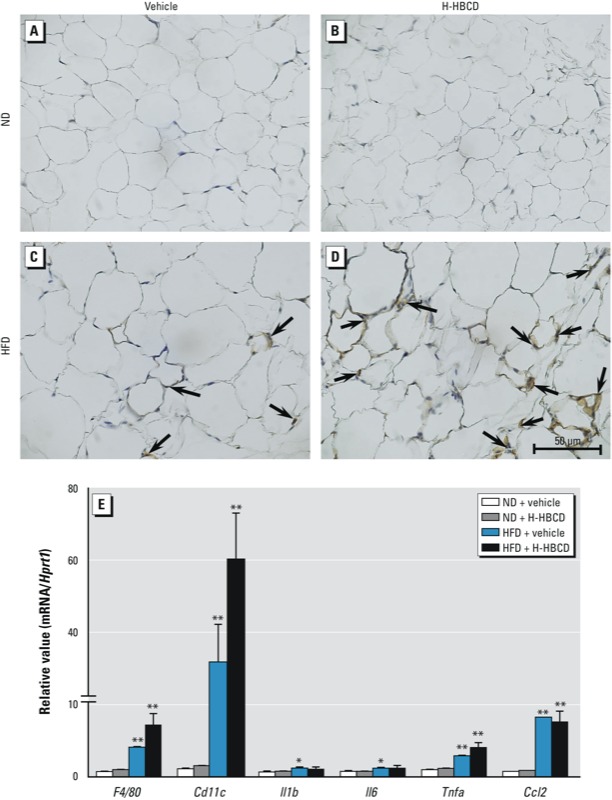
Histopathological findings and gene expression in epididymal adipose tissue 24 hr after the final administration of vehicle or HBCD. Adipocyte hypertrophy and macrophage accumulation were evaluated using F4/80 immunohistochemical staining. (*A*) ND + vehicle. (*B*) ND + H‑HBCD. (*C*) HFD + vehicle. (*D*) HBCD + H‑HBCD. Arrows indicate F4/80-positive cells. (*E*) mRNA levels of *F4/80, Cd11c, Il1b, Il6, Tnfa, and Ccl2 *in adipose tissue evaluated by RT‑PCR. Data are expressed as mean ± SE of 5–6 animals/group. Data were analyzed by Kruskal-Wallis test followed by Steel’s test. *p < 0.05, and **p < 0.01 compared with the ND + vehicle group.

*HBCD enhances adipose tissue inflammation in HFD-fed mice*. Previous studies have demonstrated that obese adipose tissue is characterized by increased infiltration of macrophages, which may be an important source of inflammation, thereby contributing to the development of metabolic disorders (Ouchi et al. 2011). To evaluate the mechanisms underlying HBCD-induced metabolic abnormality in HFD-fed mice, we examined adipocyte hypertrophy and macrophage infiltration, as revealed by *F4/80* immunostaining in epididymal adipose tissue.We observed no pathological alterations in ND-fed mice in the presence or absence of H-HBCD (Figure 2A,B). Animals fed the HFD had accentuated adipocyte hypertrophy and macrophage infiltration (Figure 2C,D). Furthermore, macrophage accumulation in the adipose tissue was more prominent in the HFD + H-HBCD group than in the HFD + vehicle group. Although not statistically significant, HBCD exposure increased mRNA levels of *F4/80* and *Cd11c* (a macrophage marker) (*p* < 0.17 for *F4/80*, *p* < 0.17 for *Cd11c*; Figure 2E). Next, we assessed proinflammatory gene expression in adipose tissue. mRNA levels of *Tnfa* (tumor necrosis factor alpha) and *Ccl2* (chemokine (C-C motif) ligand 2) in adipose tissue were higher in HFD-fed mice than in ND-fed mice. *Il1b* (interleukin 1 beta) and *Il6* (interleukin 6) mRNA were higher in the HFD + vehicle group than in the ND + vehicle group. *Tnfa* mRNA was elevated in H-HBCD–treated mice compared with vehicle-treated mice, but the difference was not statistically significant (*p* < 0.12).

**Figure 3:**
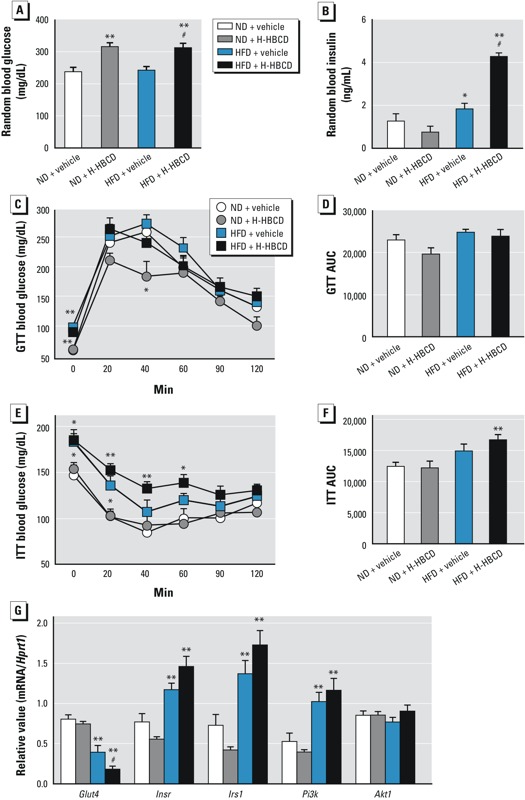
The effect of HBCD on glucose homeostasis and insulin sensitivity and on gene expression of insulin signal–related genes in epididymal adipose tissue measured 24 hr after the final administration of HBCD. (*A*) Random glucose levels. (*B*) Random insulin levels. (*C–F*) Results (mean ± SD and total area under the curve) of GTT (*C,E*) and ITT (*D,F*) performed in mice 16 and 4 hr after fasting, respectively. (*G*) mRNA levels in adipose tissue evaluated using RT‑PCR. Data are expressed as mean ± SE of 5–6 animals/group. Data were analyzed by ANOVA followed by Dunnett’s test, or by Kruskal-Wallis test followed by Steel’s test. *p < 0.05, and **p < 0.01 compared with ND + vehicle. #*p *< 0.05 compared with HFD + vehicle.

*HBCD impairs glucose homeostasis and insulin resistance in HFD-fed mice*. We evaluated serum glucose and insulin levels to determine the effect of HBCD exposure on glucose homeostasis and insulin sensitivity. We observed an increase in random glucose levels in the H-HBCD group compared with the vehicle group in both HFD- and ND-fed mice (Figure 3A). Overall, random blood insulin levels were higher in HFD mice than in ND mice, and this was more prominent in the HFD + H-HBCD than in the HFD + vehicle group (*p* < 0.05; Figure 3B). Blood glucose levels after 4 or 16 hr of fasting were much higher in HFD-fed mice than in ND-fed mice (Figure 3C,E). In the GTT, glucose tolerance was improved in the ND + H-HBCD group compared with the ND + vehicle group, whereas similar responses were observed in HFD-fed mice treated with vehicle and H-HBCD (Figure 3C). No significant differences were found in the total AUC for blood glucose levels in GTT in either group (Figure 3D). In the ITT, we observed more resistance to insulin in the HFD + HBCD group than in the HFD + vehicle group (Figure 3E). The total AUC for blood glucose levels in the ITT was significantly higher in HFD + H-HBCD mice than in ND + vehicle mice (Figure 3F).

*HBCD induces hyperglycemia and hyperinsulinemia via down-regulation of glucose transporter in HFD-fed mice*. To elucidate the mechanisms of hyperglycemia and hyperinsulinemia in HBCD-treated HFD-fed obese mice, we examined the gene expression of glucose transporter 4 (*Glut4*) in visceral adipose tissue using RT-PCR analysis (Figure 3G). mRNA levels of *Glut4* were significantly decreased in HFD-fed mice compared with ND-fed mice. *Glut4* expression was much lower in the HFD + H-HBCD group than in the HFD + vehicle group (*p* < 0.05). We then examined the effects of HBCD on the insulin-signaling pathways underlying glucose uptake in adipose tissue. mRNA levels of *Insr* (insulin receptor), *Irs1* (insulin receptor substrate 1), and *Pi3k* (phosphatidylinositol 3 kinase), but not of *Akt* (protein kinase B), were higher in HFD-fed mice than in ND-fed mice; however, H-HBCD exposure had no apparent effect on mice receiving either diet.

## Discussion

In the present study we investigated whether exposure to HBCD induces and/or enhances obesity and obesity-related metabolic dysfunction through disruption of lipid and glucose homeostasis in the liver and adipose tissue in HFD-fed and ND-fed mice. Oral HBCD treatment was associated with body weight gain, hyperglycemia, hyperinsulinemia, hepatic steatosis, and macrophage accumulation in adipose tissue in HFD-fed mice but not in ND-fed mice. In HFD-fed mice, HBCD also significantly increased *Pparg* mRNA in the liver and decreased *Glut4* mRNA in adipose tissue. These results suggest that HBCD can enhance HFD-induced weight gain and metabolic dysfunction.

This is the first study to report that orally administered HBCD appears to augment effects of HFD-induced weight gain and metabolic dysfunction but that it has no effects in mice fed a normal diet. Thus, diet-induced obese individuals may be more susceptible to HBCD than lean individuals. Regarding interactions between HFD and environmental chemicals, Wahlang et al. (2013) found that PCB-153 aggravates nonalcoholic fatty liver disease via adipokine dysregulation and altered lipid metabolism in HFD-fed mice but not in those fed a control diet. Hepatic lipid accumulation and inflammation induced by perfluorooctanoic acid (PFOA), a synthetic perfluorinated carboxylic acid, is potentiated by HFD (Tan et al. 2013). These effects may be due to the highly persistent and lipophilic properties of environmental chemicals in obese individuals, and this greater body burden of HBCD in HFD-induced obesity may enhance weight gain and metabolic dysfunction.

Several studies have suggested that females are more susceptible to HBCD than males. Cantón et al. (2008) reported that subacute exposure to HBCD down-regulated cholesterol biosynthesis– and lipid metabolism–related gene expression in female rat liver, but up-regulated drug-metabolizing enzyme-related gene expression in male rat liver. Oral exposure to HBCD may induce drug-metabolizing enzymes more prominently in female rats than in males (Germer et al. 2006). In the present study, HBCD-exposed HFD-fed male mice had increased hepatic steatosis and hepatic TG levels (Figure 1D,E), but no significant effects were seen in ND-fed mice. Thus, the obesogenic effects of HBCD in males may depend on the fat in the diet. Different findings among studies may be also explained by differences in concentration and duration of HBCD exposure; further studies are required to define the effects of HBCD, particularly in female mice.

In the present study, HBCD-treated HFD-fed mice had increased random blood glucose and random blood insulin, and a tendency toward impaired insulin resistance. Furthermore, we found a significant decrease in *Glut4* mRNA levels in adipose tissue in HFD-fed HBCD-treated mice. GLUT4, an insulin-sensitive glucose transporter found in adipose tissue, skeletal muscle, and the heart, plays a critical role in glucose homeostasis and functions as a key modulator of glucose disposal in fat (Watson et al. 2004). Decreased insulin sensitivity has been reported in *Glut4-*null mice (Carvalho et al. 2005). In obesity, decreased *Glut4* gene expression is directly related to the development of human insulin resistance (Garvey et al. 1998). Inflammatory molecules generated in adipose tissue, such as TNF-α and IL-6, are related to decreased GLUT4 expression (Rotter et al. 2003), which leads to insulin resistance due to decreased adipocyte glucose uptake (Leguisamo et al. 2012). In the present study, although not statistically significant, *Tnfa* mRNA increased in the adipose tissue of HBCD-treated HFD-fed mice. Decreased expression of GLUT4 might, in part, be explained by TNF-α expression. These results suggest that HBCD in HFD-fed mice may decrease glucose transport, resulting in hyperinsulinemia and hyperglycemia. Fat accumulation in the liver and insulin resistance may also enhance metabolic dysfunction and development of hepatic steatosis (Shang et al. 2008). In HFD-fed mice in the present study, we observed prominent hepatic steatosis and elevated hepatic TG levels in HBCD-treated compared with vehicle-treated mice. These findings indicate that HBCD plus HFD may have a role in the disruption of lipid and glucose metabolism in the liver and adipose tissue.

In the present study, we also observed elevated hepatic mRNA levels of *Pparg* and *Fsp27* in HBCD-treated HFD-fed mice compared with vehicle-treated HFD-fed mice. Although not statistically significant, similar patterns were observed for target genes of PPARγ, such as *Cd36* and *Fabp4* (Figure 1G). PPARγ promotes lipogenesis and adipogenesis in adipose tissue (Wan et al. 2007) and is normally expressed at low levels in human and mouse liver (10–30% of adipose tissue) (Semple et al. 2006). However, HFD induces hepatic PPARγ expression accompanied by hepatic steatosis (Inoue et al. 2005). Schadinger et al. (2005) showed that PPARγ in the liver of ob/ob mice (a murine model of type 2 diabetes) induced lipid accumulation in hepatocytes. *Cd36*, *Fabp4*, and *Fsp27* are target genes of PPARγ involved in fatty acid transportation (Greco et al. 2008) and fat droplet deposition in the liver (Matsusue et al. 2008). CD36 is a membrane receptor associated with uptake of oxidized low-density lipoproteins (Endemann et al. 1993) and mediates hepatic fatty acid uptake, which induces hepatic steatosis (Bradbury 2006). Shearer et al. (2005) found that *Fabp4*-null mice are protected against diet-induced obesity, insulin resistance, and fatty liver. Fsp27 is a lipid droplet-binding protein that promotes lipid accumulation in adipocytes. A recent study showed that *Fsp27* in the liver of ob/ob mice is a direct target gene of PPARγ and can elevate hepatic TG levels (Matsusue et al. 2008). In contrast, in the present study, we observed no changes in expression of lipogenesis-related genes such as *Fasn* and *Srebp1* in either diet group. These results suggest that lipogenesis may not underlie HBCD-induced hepatic TG accumulation. Taken together, these results suggest that HBCD exposure in HFD-fed mice activates PPARγ and, possibly, lipid transport-related genes induced by PPARγ, and that these changes lead to development of hepatic steatosis.

Chronic and low-grade inflammation are causes of obesity resulting in metabolic dysfunction (Ouchi et al. 2011). Several studies have shown an important role of adipose tissue macrophages in inflammation in obesity through the production and release of proinflammatory mediators such as IL-1, TNF-α, IL-6, and CCL2 (Hrnciar et al. 1999; Xu et al. 2003). IL-1β and TNF-α are secreted from macrophage and adipocyte inflammation. CCL2 plays a critical role in macrophage accumulation and activation. CCL2 expression in adipose tissue induces TNF-α generation through interactions between macrophages and adipocytes (Suganami et al. 2005), thereby blocking insulin signaling in adipocytes (Hotamisligil 2006). In the present study, HFD + H-HBCD mice had increased mRNA levels of *F4/80, Cd11c*, and *Tnfa* (although not statistically significant) and increased macrophage accumulation in adipose tissue compared with HFD + vehicle mice (Figure 2E). These findings suggest that HBCD exposure in diet-induced obesity may accelerate adipose tissue inflammation due to increased accumulation of macrophages and, possibly, proinflammatory mediators derived from macrophages.

HBCD dietary intake in humans has been estimated to be 141–151 ng/day in Sweden (Lind et al. 2002) and 350–410 ng/day in the United Kingdom (Food Standards Agency 2006). Given the persistent and lipophilic properties of HBCD, there is a need for further investigation of the long-term health effects of chronic low-dose exposure to HBCD *in vivo*. These data may support the hypothesis that HBCD exposure may accelerate the obesity epidemic in humans.

## Conclusions

The present study showed that enhanced weight gain, hyperglycemia, hyperinsulinemia, hepatic steatosis, and macrophage accumulation in adipose tissue in HBCD-treated HFD-fed mice but not in HBCD-treated ND-fed mice. These results suggest that HBCD may contribute to metabolic dysfunction via an interaction with diet (i.e., HBCD may be an “enhancer” obesogen). We found that HBCD contributes to the progression of diet-induced weight gain and metabolic dysfunction, suggesting that HBCD may increase the risk of diet-induced obesity.

## Supplemental Material

(119 KB) PDF
