# Supplementary material for: Impaired Lipid and Glucose Homeostasis in Hexabromocyclododecane-Exposed Mice Fed a High-Fat Diet
Source: Environ Health Perspect. 2014 Jan 7;122(3):277–83. doi: 10.1289/ehp.1307421 (PMC3948039; doi:10.1289/ehp.1307421)
Supplement: (119 KB) PDF [file ehp.1307421.s001.pdf]

Environmental Health Perspectives doi:10.1289/ehp.1307421

Supplemental Material

Impaired Lipid and Glucose Homeostasis in Hexabromocyclododecane-Exposed Mice Fed a High-Fat Diet

Rie Yanagisawa, Eiko Koike, Tin-Tin Win-Shwe, Megumi Yamamoto, and Hirohisa Takano

Table of Contents

Supplemental Material, Table S1. The composition of normal-diet and high-fat diet page 2

Supplemental Material, Table S2. Gene list for RT-PCR analysis page 3

Supplemental Material, Table S3. Body and liver weight and biochemical test in the serum at 20 weeks of age page 4

Supplemental Material, Figure S1. Chemical structure of hexabromocyclododecane (HBCD) page 5

Supplemental Material, Figure S2. Effects of HBCD exposure on body weight gain in the ND- or HFD-fed mice page 6

Supplemental Material, Table S1. The composition of normal-diet and high-fat diet.

	**AIN-93M (g/100 g)**	**HFD-60 (g/100 g)**
Milk casein	14.00	25.60
L-cysteine	0.18	0.36
Corn starch	46.57	-
α-Corn starch	15.50	16.00
Maltodextrin	-	6.00
Sucrose	10.00	5.50
Soybean oil	4.00	2.00
Powdered cellulose	5.00	6.61
AIN-93M mineral mixture	3.50	3.50
AIN-93M vitamin mixture	1.00	1.00
Choline bitartrate	0.25	0.25
Calcium carbonate	-	0.18
Lard	-	33.00
tert-butylhydroquinone	0.0008	-
Total calorie (kcal/100g)	360.5	506.2

Supplemental Material, Table S2. Gene list for RT-PCR analysis.

Category	Gene symbol	Gene name	Assay ID
Transcription factor	*Ppara*	peroxisome proliferator activated receptor alpha	Mm00440939_m1
Transcription factor	*Pparg*	peroxisome proliferator activated receptor beta	Mm01184322_m1
Transcription factor	*LXR*	liver X receptor	Mm00443451_m1
Transcription factor	*Rxra*	retinoid X receptor alpha	Mm00441182_m1
Transcription factor	*Srebp1*	sterol regulatory element binding transcription protein 1	Mm00550338_m1
Lipid transport	*Cd36*	CD36 antigen	Mm01135198_m1
Lipid transport	*Fabp4*	fatty acid binding protein 4	Mm00445878_m1
Lipid transport	*Fsp27*	fat-specific protein 27	Mm00617672_m1
Lipogenesis	*Fasn*	fatty acid synthase	Mm00662319_m1
Macrophage marker	*F4/80*	F4/80	Mm00802529_m1
Macrophage marker	*Cd11c*	integrin alpha X	Mm00498698_m1
Inflammation	*Il1b*	interleukin 1 beta	Mm00434228_m1
Inflammation	*Il6*	interleukin 6	Mm00446190_m1
Inflammation	*Tnfa*	tumor necrosis factor alpha	Mm00443260_g1
Inflammation	*Ccl2*	chemokine (C-C motif) ligand 2	Mm00441242_m1
Insulin signaling	*Insr*	insulin receptor	Mm01211875_m1
Insulin signaling	*Irs1*	insulin receptor substrate 1	Mm01278327_m1
Insulin signaling	*Pi3k*	phosphatidylinositol 3-kinase	Mm00803160_m1
Insulin signaling	*Akt1*	protein kinase B	Mm01331626_m1
Insulin signaling	*Glut4*	glucose transporter 4	Mm01245502_m1Gl

RT-PCR; reverse transcription polymerase chain reaction.

Supplemental Material, Table S3. Body and liver weight and biochemical test in the serum at 20 weeks of age.

Group	Body weight gain (g)	Liver weight (mg)	AST (IU/L)	ALT (IU/L)	T-Cho (mg/dL)	TG (mg/dL)
ND+Vehicle	8.94 ± 0.61	1261 ± 54.8	73.0 ± 8.86	13.6 ± 1.04	136 ± 8.39	86.1 ± 9.88
ND+L-HBCD	9.07 ± 0.70	1283 ± 36.8	74.2 ± 7.59	15.0 ± 1.18	147 ± 7.54	81.8 ± 4.72
ND+M-HBCD	8.53 ± 0.51	1159 ± 21.9	66.6 ± 6.57	14.2 ± 1.59	133 ± 6.53	93.6 ± 3.93
ND+H-HBCD	7.74 ± 1.13	1165 ± 49.4	46.0 ± 7.96*	10.5 ± 0.22	116 ± 10.6	89.8 ± 13.1
HFD+Vehicle	15.8 ± 1.50**	1405 ± 96.4**	79.7 ± 7.44	34.5 ± 8.43	202 ± 5.89**	90.5 ± 9.59
HFD+L-HBCD	17.7 ± 1.83**	1622 ± 164**	78.7 ± 8.58	43.0 ± 15.0	200 ± 7.29**	87.7 ± 6.37
HFD+M-HBCD	20.8 ± 0.97$^{**,\#}$	1662 ± 87.9$^{**,\#\#}$	101 ± 8.39*	60.0 ± 12.2**	220 ± 5.07**	96.2 ± 10.4
HFD+H-HBCD	21.3 ± 1.31$^{**,\#\#}$	1790 ± 153$^{**,\#\#}$	85.2 ± 7.50	61.5 ± 10.2**	212 ± 1.95**	98.3 ± 8.11

Serum levels of aspartate aminotransferase (AST), alanine animotransferase (ALT), total cholesterol (T-Cho), triglyceride (TG), and glucose 24 hr after the final HBCD administration were measured by SPOTCHEM EZ SP-4430 (ARKRAY, Inc., Kyoto, Japan). ALT; alanine animotransferase, AST; aspartate aminotransferase, T-Cho; total cholesterol, TG; triglyceride. Data are the mean ± SE of 5-6 animals per group. Data were analyzed ANOVA followed by Dunnett's test or Kruskal-Wallis followed by Steel's test. $^{*}P < 0.05$ vs. ND+Vehicle group, $^{**}P < 0.01$ vs. ND+Vehicle group, $^{\#}P < 0.05$ vs. HFD+Vehicle group, $^{\#\#}P < 0.01$ vs. HFD+Vehicle group.

Supplemental Material, Figure S1.

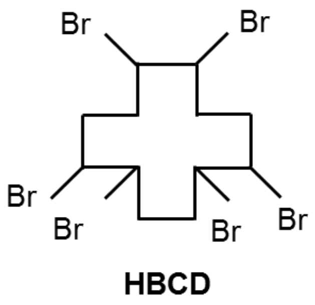

Figure S1. Chemical structure of hexabromocyclododecane (HBCD)

Supplemental Material, Figure S2

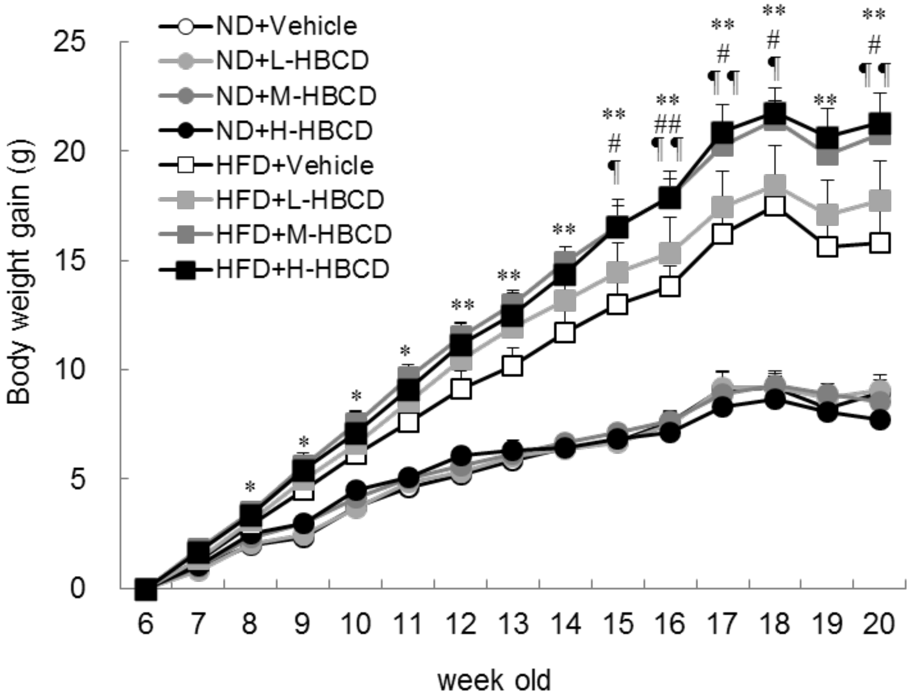

Figure S2. Effects of HBCD exposure on body weight gain in the ND- or HFD-fed mice.

Data are the mean ± SE of 5-6 animals per group. Data were analyzed ANOVA followed by Dunnett's test or Kruskal-Wallis followed by Steel's test.

The results for ND + Vehicle group were not visible in the figure because of overlap with other data points.

*; $P < 0.05$ vs. ND+Vehicle group, ** $P < 0.01$ vs. ND+Vehicle group, # $P < 0.05$ HFD+M-HBCD group vs. HFD+Vehicle group, ## $P < 0.01$ HFD+M-HBCD group vs. HFD+Vehicle group, ¶ $P < 0.05$ HFD+H-HBCD group vs. HFD+Vehicle group, ¶¶ $P < 0.01$ HFD+H-HBCD group vs. HFD+Vehicle group.
